# A Forgotten Encounter: A Case of Amebic Liver Abscess Mimicking Hepatic Malignancy in a Non-endemic Region

**DOI:** 10.7759/cureus.76913

**Published:** 2025-01-04

**Authors:** Byron Lee, Tejasvi Ayyagari, Zheng Song, Stanley Kim

**Affiliations:** 1 Hematology and Medical Oncology, Western University of Health Sciences, College of Osteopathic Medicine of the Pacific-Northwest, Lebanon, USA; 2 Internal Medicine, Ross University School of Medicine, Miramar, USA; 3 Family Medicine, Rio Bravo Family Medicine Residency Program, Bakersfield, USA; 4 Hematology and Oncology, Kern Medical, Bakersfield, USA

**Keywords:** amebic liver abscess, diagnosis of amebic liver abscess, e. histolytica, entamoeba histolytica, liver mass, radiological findings of amebic liver abscess

## Abstract

Amebic liver abscess (ALA), caused by *Entamoeba histolytica*, is prevalent in endemic regions such as Central/South America, Southeast Asia/India, and Africa but is considered rare in North America. Symptoms of *E. histolytica* infection typically emerge eight to 20 weeks after exposure in endemic areas.

We present the case of a 58-year-old woman who developed right-sided abdominal pain, weakness, fever, and significant weight loss. Radiological imaging revealed a large, non-cystic hepatic mass, raising suspicion of malignancy. Notably, the patient had not traveled to an endemic region in the past year.

An ultrasound-guided liver biopsy drained 500 mL of purulent, grayish fluid. Cultures for bacteria and cytology for malignancy were negative. Further serological testing confirmed the diagnosis of ALA with positive *E. histolytica* IgG antibodies. The patient was treated with oral metronidazole, resulting in rapid symptomatic improvement.

This case underscores the diagnostic challenges associated with ALA in non-endemic regions, particularly when initial presentation, imaging findings, and absence of recent travel history suggest hepatic malignancy. It highlights the importance of considering ALA in the differential diagnosis of hepatic masses, even in patients without clear exposure to endemic regions.

## Introduction

Amebic liver abscess (ALA) is caused by the protozoan parasite *Entamoeba histolytica (E. histolytica)*. Transmission typically occurs through fecally contaminated food or water. It is a rare condition in non-endemic areas, such as North America, but highly prevalent in countries of Central America, South America, Southeast Asia, the Indian continent, and Africa [1]. Although most infections with *E. histolytica* are asymptomatic, amebic dysentery is the most common presentation among symptomatic infections [1]. Symptoms of *E. histolytica* infections usually emerge within eight to 20 weeks after exposure in endemic regions [2]. Rarely, it can cause extraintestinal disease when the trophozoite invades the intestinal mucosa and disseminates through the bloodstream [1,3]. ALAs are the most common extraintestinal presentation as the trophozoites reach the liver via portal venous circulation [1,3]. ALA can mimic liver malignancy, both clinically and radiologically. Patients often develop chronic fatigue, weight loss, and fever, which are common symptoms of malignancy. Ultrasound (US) typically shows hypoechoic lesions without any significant wall echoes, and contrast-enhanced computerized tomography (CT) usually demonstrates round-shaped, hypoattenuating lesions with an enhancing wall and a peripheral edema [4]. Antibodies against *E. histolytica* are detectable at presentation in 92%‐97% of patients with amebic liver abscesses. Aspiration of amebic liver abscesses is not necessary for establishing the diagnosis, as trophozoites are seen in a minority (<20%) of aspirates [1]. Metronidazole for seven to 10 days is the treatment of choice with a cure rate of >90% [1,3]. Here, we present a patient with a large liver lesion mimicking malignancy, which turned out to be ALA.

## Case presentation

A 58-year-old Hispanic female with a distant history of coccidioidomycosis diagnosed 20 years ago was admitted to Kern Medical Center through the emergency department (ED) with a one-month history of progressive right-sided chest pain and dyspnea. Her symptoms were accompanied by unintended weight loss of 15 pounds over the past month, intermittent subjective fevers, and chills for one week. Her family noted that she appeared increasingly frail, with a slow gait and significant weakness. Four months prior to admission, the patient attended the Kern County Fair, where she consumed nacho chips with cheese. This was followed by a one-month episode of diarrhea, after which her current symptoms gradually developed. She also reported a history of travel to Mexico one year ago. On admission, the patient presented with the following vital signs: blood pressure (BP) 100/60 mmHg, temperature 38.2°C, pulse 103/minute, and respiratory rate 28/minute. She was not in respiratory distress but had diminished breath sounds in the right lower lung and mild tenderness in the right upper quadrant of the abdomen.

Laboratory results (Table 1) revealed elevated alkaline phosphatase (215 U/L), erythrocyte sedimentation rate (95 mm/h), and C-reactive protein (10.4 mg/dL); hemoglobin (Hb: 9.8 g/dL); normal white blood cell (WBC) count: 7.7 × 10³/mcL; normal transaminases and bilirubin levels; normal creatinine level. Serology tests for coccidioidomycosis, HIV, syphilis, and hepatitis B and C were negative.

**Table 1:** Patient’s laboratory values on admission ESR: erythrocyte sedimentation rate

Laboratory Test	Patient’s Value	Normal Range
Alkaline Phosphatase	215 U/L	45-117
Albumin	2.5 g/dL	3.4-5.0
C-reactive Protein	10.4 mg/dL	<=0.30
White Blood Cell	7.7 x 103/mcL	4.5-11
Hemoglobin	9.8 g/dL	11.1-15.4
Platelet	554 x 103/mcL	150-450
ESR	95 mm/hr	<=30

**Figure 1:**
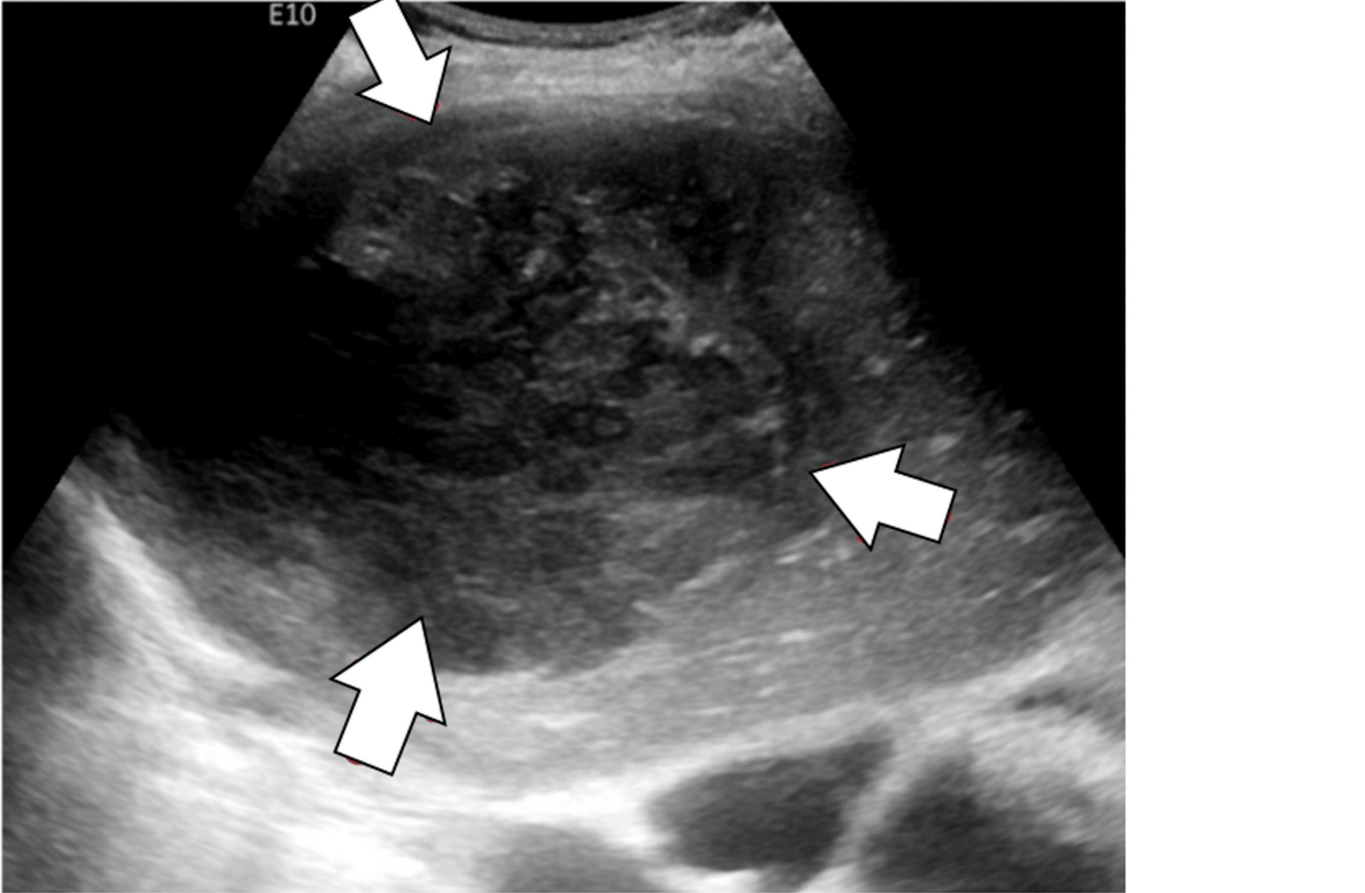
Ultrasound of the abdomen identified a solid mass within the right hepatic lobe near the dome of the liver measuring 12.9 x 9.8 x 9.6 cm, concerning for underlying neoplasm

A CT chest scan showed an elevated right hemidiaphragm with a heterogeneous 12 cm mass in the right hepatic lobe. An abdominal ultrasound (Figure 1) revealed a solid mass in the right hepatic lobe near the dome of the liver measuring 12.9 × 9.8 × 9.6 cm, raising concerns for malignancy.

**Figure 2:**
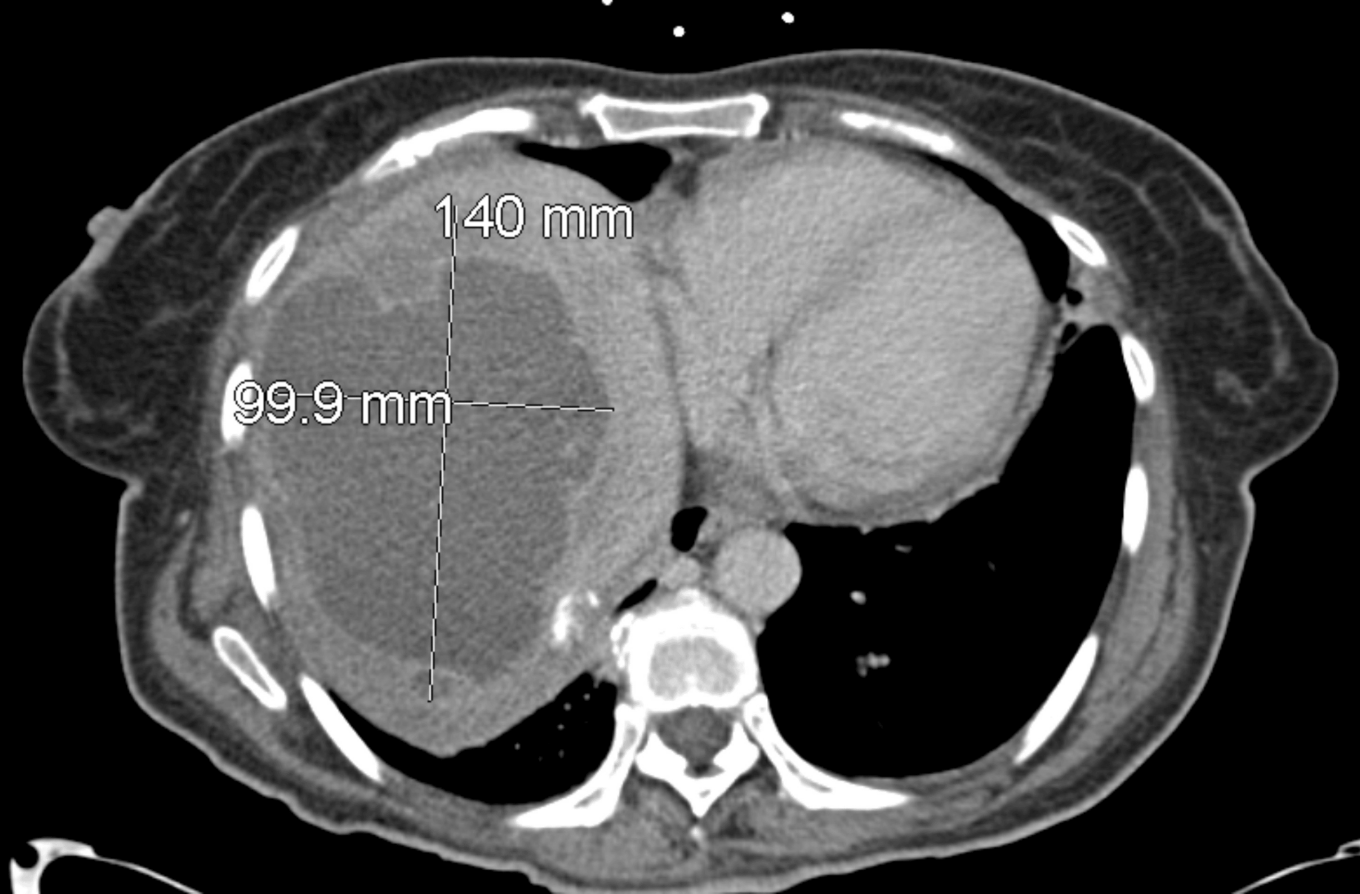
CT abdomen and pelvis revealing a 14 x 10 x 13 cm heterogeneous mass localized within the right hepatic lobe

A subsequent CT abdomen and pelvis with contrast (Figure 2) confirmed a lobulated, hypodense, non-cystic lesion measuring 14 × 10 × 13 cm. 

**Figure 3:**
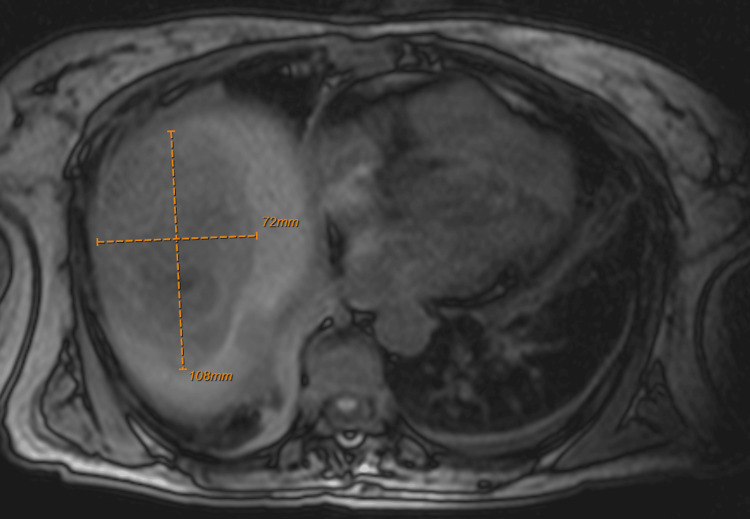
MRI abdomen showing a reduction in the size of the mass

Tumor markers (AFP, CA 19-9, and CEA) were normal. Hydatid cysts were initially considered but ruled out based on the absence of cystic features on imaging. The patient underwent an ultrasound-guided liver biopsy, yielding approximately 500 mL of grayish purulent fluid. A drainage catheter was placed. Immediately post-procedure, she experienced a transient episode of hypotension and syncope, which resolved with intravenous fluids. Cultures of the aspirate and blood were negative for bacterial growth. Magnetic resonance imaging (Figure 3) performed two days after drainage showed a reduction in lesion size from 14 × 10 × 13 cm to 10.8 × 7.2 × 9.6 cm.

Given her travel history and clinical presentation, serology for *Entamoeba histolytica* was performed and came back positive for *E. histolytica* IgG antibodies. Based on the serological results, clinical symptoms, and imaging findings, a diagnosis of amebic liver abscess (ALA) was made.

The patient was started on oral metronidazole 750 mg three times daily, which led to rapid improvement in fever, weakness, and anorexia. She was hospitalized for ten days and discharged with continued oral metronidazole.

## Discussion

An amebic liver abscess (ALA) is caused by *Entamoeba histolytica*, a protozoan parasite primarily transmitted through ingestion of fecally contaminated food or water [1]. While rare in the United States, ALA remains a significant concern for immigrants or travelers from endemic regions. Infected individuals typically develop symptoms within eight to 20 weeks after returning from endemic areas (median: 12 weeks) [2]. However, in some cases, the latency period can be significantly prolonged, with reports of ALA developing up to 32 years post-infection [5].

*E. histolytica *infection often begins as asymptomatic colonization within the enterocytes of the gastrointestinal tract. The parasite can disseminate to extraintestinal sites via the portal circulation, with the liver being the most common site of infection [1,2]. In our patient, a one-month episode of diarrhea following her visit to the Kern County Fair raised concerns about recent exposure to contaminated food. However, the possibility of reactivation from a latent asymptomatic infection acquired during her travel to Mexico one year earlier cannot be excluded.

ALA can mimic hepatic malignancy both clinically and radiologically. Common symptoms such as right-sided abdominal pain, weakness, fever, and weight loss overlap significantly with presentations of hepatic malignancies [6]. Radiologically, ALA can resemble malignant liver tumors. Ultrasound findings typically include round or oval hypoechoic lesions without significant wall echoes, while contrast-enhanced CT scans often show hypoattenuating, ill-defined lesions with peripheral edema and an enhancing wall [4]. In this case, the patient’s initial CT scan revealed a large, non-cystic hepatic mass suspicious of malignancy. However, tumor markers commonly associated with liver malignancies (AFP, CA 19-9, and CEA) were negative, prompting further investigation.

In non-endemic settings, pyogenic liver abscess is a key differential diagnosis for ALA. Pyogenic abscesses are typically characterized by positive blood or aspirate cultures and are often multiple and smaller in size [7,8]. Our patient, however, presented with a single large abscess localized to the right hepatic lobe, with negative bacterial cultures from both blood and aspirate, which is more consistent with ALA. Additionally, the anemia and elevated alkaline phosphatase levels observed in our patient have been reported in indolent ALA cases, particularly in non-endemic areas [7].

On the other hand, primary liver malignancy, such as intrahepatic cholangiocarcinoma, may present as a non-resolving liver abscess [9]. Therefore, a longer follow-up is necessary to ensure the complete resolution of liver abscess.

The low prevalence of ALA in the United States and the potential for prolonged latency often result in delayed diagnosis and treatment. Although ALA development one year after travel to an endemic region is uncommon, it should remain on the differential diagnosis. A detailed social and travel history is crucial for early suspicion and timely management of ALA. Amoebic serology remains a reliable diagnostic tool for confirmation in such cases.

## Conclusions

This case underscores the diagnostic challenges associated with ALA in non-endemic regions, particularly when initial presentation, imaging findings, and absence of recent travel history suggest hepatic malignancy. It highlights the importance of considering ALA in the differential diagnosis of hepatic masses, even in patients without clear exposure to endemic regions for prompt recognition and appropriate treatment.

## References

[1] Anesi JA, Gluckman S (2015). Amebic liver abscess. Clin Liver Dis (Hoboken).

[2] Broz P, Jacob AL, Fehr J, Kissel CK (2010). An unusual presentation of amebic liver abscesses. CMAJ.

[3] Haque R, Huston CD, Hughes M, Houpt E, Petri WA Jr (2003). Amebiasis. N Engl J Med.

[4] Karaosmanoglu AD, Uysal A, Karcaaltincaba M, Akata D, Ozmen MN, Kraeft J, Hahn PF (2020). Non-neoplastic hepatopancreatobiliary lesions simulating malignancy: can we differentiate?. Insights Imaging.

[5] Nespola B, Betz V, Brunet J (2015). First case of amebic liver abscess 22 years after the first occurrence. Parasite.

[6] Sun VC, Sarna L (2008). Symptom management in hepatocellular carcinoma. Clin J Oncol Nurs.

[7] Wuerz T, Kane JB, Boggild AK (2012). A review of amoebic liver abscess for clinicians in a nonendemic setting. Can J Gastroenterol.

[8] Lodhi S, Sarwari AR, Muzammil M, Salam A, Smego RA (2004). Features distinguishing amoebic from pyogenic liver abscess: a review of 577 adult cases. Trop Med Int Health.

[9] Shah V, Arora A, Tyagi P (2015). Intrahepatic cholangiocarcinoma masquerading as liver abscess. J Clin Exp Hepatol.

